# Systematic review of team performance in minimally invasive abdominal surgery

**DOI:** 10.1002/bjs5.50133

**Published:** 2019-01-30

**Authors:** W. J. van der Vliet, S. M. Haenen, M. Solis‐Velasco, C. H. C. Dejong, U. P. Neumann, A. J. Moser, R. M. van Dam

**Affiliations:** ^1^ Department of Hepatobiliary and Pancreatic Surgery, Maastricht University Medical Centre, Maastricht, the Netherlands; ^2^ Pancreas and Liver Institute, Beth Israel Deaconess Medical Centre, Harvard Medical School, Boston, Massachusetts, USA; ^3^ Department of Surgery, Universitätsklinikum Aachen, Aachen, Germany

## Abstract

**Background:**

Adverse events in the operating theatre related to non‐technical skills and teamwork are still an issue. The influence of minimally invasive techniques on team performance and subsequent impact on patient safety remains unclear. The aim of this review was to assess the methodology used to objectify and rate team performance in minimally invasive abdominal surgery.

**Methods:**

A systematic literature search was conducted according to the PRISMA guidelines. Studies on assessment of surgical team performance or non‐technical skills of the surgical team in the setting of minimally invasive abdominal surgery were included. Study aim, methodology, results and conclusion were extracted for qualitative synthesis.

**Results:**

Sixteen studies involving 677 surgical procedures were included. All studies consisted of observational case series that used heterogeneous methodologies to assess team performance and were of low methodological quality. The most commonly used team performance objectification tools were ‘construct’‐ and ‘incident’‐based tools. Evidence of validity for the assessed outcome was spread widely across objectification tools, ranging from low to high. Diverse and poorly defined outcomes were reported.

**Conclusion:**

Team demands for minimally invasive approaches to abdominal procedures remain unclear. The current literature consists of studies with heterogeneous methodology and poorly defined outcomes.

## Introduction

A substantial contribution to morbidity among surgical patients can be attributed to adverse events occurring in the operating theatre^1^. Increasing evidence shows that a considerable portion of these adverse events cannot be attributed solely to deficient technical skills^2^, ^3^, ^4^. Adverse events related to non‐technical skills and team performance are common and estimated to be twice as frequent as errors in surgical technique^5^. Poor teamwork and lack of vigilance appear to be essential factors influencing procedural flow and increasing error rates^6^.

Surgical teams demand specific infrastructure, resources and competencies to perform effectively and maintain patient safety^7^. Effective team performance depends on physical and social interactions, including back‐up behaviour and leadership^8^. These demands, competencies and interactions encompassing effective team performance create a domain that is difficult to objectify and quantify^7^, ^9^.

In recent years, minimally invasive techniques have become the benchmark for a large number of abdominal surgical procedures^10^. These approaches introduce complex equipment, increased numbers of instrument changes and larger teams to the operative environment, resulting in increased demands in levels of coordination, anticipation, planning and communication^11^. The impact of the variation in procedural approaches on team demands, error rates and patient safety remains unclear. In highly complex abdominal procedures, associated with learning curves for surgical technique, minimally invasive approaches could also have a significant impact on team performance and non‐technical skills^12^.

Recent studies have used a variety of methodologies to observe and objectify team performance. Consensus on the most efficient and methodically correct way of analysing and rating effective team performance is yet to be reached. The development of benchmarks for team observations and assessment of team performance will allow an accurate comparison of demands relative to surgical techniques. This will facilitate the development of effective, evidence‐based training programmes for surgical teams, directed to increase team performance, decrease error rates and increase patient safety.

The aim of this systematic review was to assess the methodology used to objectify surgical team performance in minimally invasive abdominal surgery and explore team demands in relation to non‐technical skills.

## Methods

This study was performed according to the PRISMA guidelines^13^. Two researchers were involved in the search, inclusion, critical appraisal and data extraction of the articles selected for this study.

### Eligibility criteria

Studies on assessment of surgical team performance and non‐technical skills of the entire surgical team (including surgeons, anaesthesia and nursing staff) in the setting of minimally invasive abdominal surgery were included. Abdominal surgery was defined as any urological, gynaecological or general surgical procedure performed intra‐abdominally. Minimally invasive techniques consisted of minimal‐access approaches to the abdominal cavity, including laparoscopic, video‐ or robot‐assisted methods.

Exclusion criteria consisted of non‐original research, research performed in a simulated environment or non‐human subject research, and language of publication other than English.

### Study selection

Two authors performed a systematic literature search using PubMed, Embase, the Cochrane Library and Google Scholar to identify articles published before 11 October 2017. Search terms were based on subject (‘teamwork’, ‘team learning’, ‘team efficiency’, ‘non‐technical skills’) and setting (‘minimally invasive abdominal surgery’, ‘laparoscopic surgery’, ‘robotic surgery’). After the initial search, duplicates and non‐English studies were removed. Articles were screened for eligibility by title, abstract and then full text. Reference lists and citations of the included studies were screened for missed articles. Discrepancies in study selection between the two authors were discussed with other review team members until consensus was reached.

### Critical appraisal

The methodological quality of the included studies was assessed using the Oxford Centre for Evidence‐Based Medicine levels of evidence, ranging from 1 (systematic review of RCTs) to 5 (expert opinion)^14^. Evidence of validity of the tools used to objectify and rate operative team performance was assessed using Messick's framework^15^, where a test should be rated for construct validity in each specific context in which the test is employed, defining five sources of evidence (content, process response, internal structure, relations to other variables, and consequences) each rated on a three‐point scale^16^. Evidence of validity was classified as low (0–5), moderate (6–10) or high (11–15).

### Data collection

The following data were extracted from the included studies: aim, design, setting, studied procedures, observational method, observer characteristics, outcomes, conclusion, and assessment of team performance and non‐technical skills.

## Results

**Figure 1:**
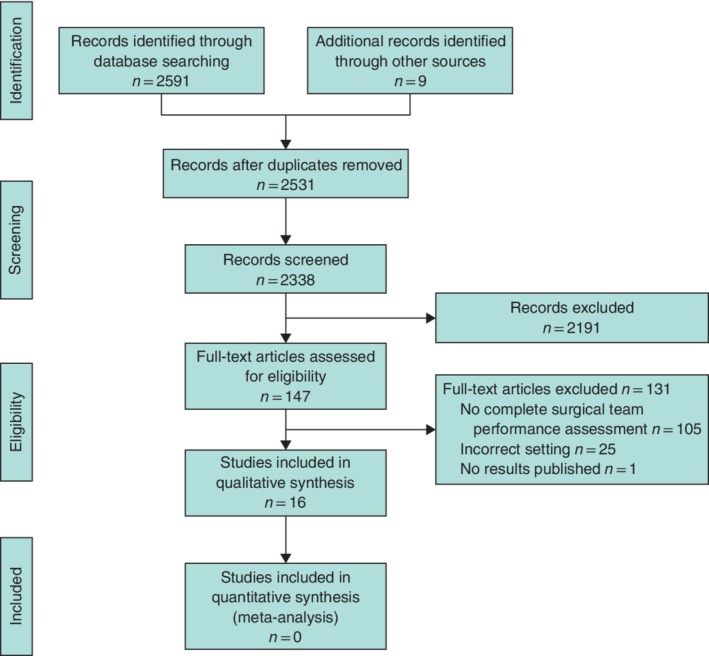
Flow chart showing selection of articles for review

The systematic search identified 2591 manuscripts, from which 69 duplicates and 193 non‐English publications were removed. Based on screening of title and abstract a further 2198 articles were excluded. The remaining 138 full‐text publications were reviewed, resulting in the selection of seven studies. Subsequent review of citations and references lists led to the inclusion of a further nine articles, so that a total of 16 studies were finally included in this systematic review (*Fig*. 1).

### Study aims, designs and settings

All included studies consisted of single‐centre observational series, with the exception of one dual‐centre series^17^ of four robot‐assisted procedures. Two studies^18^, ^19^ investigated the influence of a non‐technical skills training intervention on surgical team performance. Four studies^19^, ^20^, ^21^, ^22^ consisted of subanalyses of results from observed cohorts published in previous work (*Table* S1, supporting information).

According to the Oxford Centre for Evidence‐Based Medicine, all studies provided level 4 evidence (case series, poor‐quality cohort studies, case–control studies). No subgroup analysis or meta‐analysis of outcomes data was attempted due to heterogeneous methodologies and outcome measurements across the included studies.

All studies observed complete surgical teams (including surgery, anaesthesia and nursing staff) with the goal of evaluating team performance through surgical workflow analysis and evaluation of disruptive events (9 studies)^20^, ^23^, ^24^, ^25^, ^26^, ^27^, ^28^, ^29^, ^30^, the relationship between team performance and technical outcomes (3 studies)^18^, ^21^, ^22^, the relationship between anticipation of surgical steps and team efficiency (1 study)^31^ or novel tools to rate team performance (3 studies)^17^, ^19^, ^32^.

Of the 16 studies, 11 focused exclusively on minimally invasive procedures, whereas five included both open and minimally invasive approaches to abdominal surgical procedures. Ten studies investigated laparoscopic techniques and six a robot‐assisted approach. A total of 677 procedures (281 laparoscopic, 236 robot‐assisted and 160 open) were observed across the included studies, with a mean of 42.3 operations per study (*Table* S1, supporting information).

### Observational methodology

The majority of studies (12) observed team performance directly, and the four remaining studies performed a postoperative review of audiovisual recordings. Most (13) used multiple observers to evaluate team performance, with 14 reporting on methodological training of observers before the study and five including experts trained in human factor assessment or psychologists in their observing teams. Nine studies quantified interobserver reliability using a variety of methodologies; reliability was deemed good to excellent (*Table* S1, supporting information).

Most studies (14) observed team performance for the entire duration of the patient being present in the operating theatre. Six studies subdivided the procedure into preoperative, intraoperative and postoperative phases, of which four also defined a robot‐docking phase.

### Assessment of team performance

Seven studies used ‘construct‐based’ team performance assessment tools that rated a number of behaviour constructs to create an overall score at the end of a case. Construct‐based tools included: Oxford Non‐Technical Skills (NOTECHS)^19^ (4 studies) or Observational Teamwork Assessment for Surgery (OTAS)^33^ (3 studies). These tools contained moderate evidence of validity for the assessed outcome according to Messick's framework, with a range of 8–10 of 15 (*Table* 1). Nine studies used an ‘incident‐based’ team performance assessment methodology, classifying non‐technical procedural errors or disruptions of surgical flow in order of causation^34^. The evidence of validity for these outcome tools was spread widely, ranging from low to high (4–12 of 15) (*Table* 1).

**Table 1:** Outcome assessment tool validity according to Messick's framework of validity

	Source of validity					
Outcome assessment tool	Content	Response process	Internal structure	Relation to other variables	Consequence	Total
**Construct**						
NOTECHS						
Catchpole *et al*.^21^	3	3	0	2	1	9
Mishra *et al*.^22^	3	2	1	1	1	8
McCulloch *et al*.^18^	3	3	1	1	1	9
Mishra *et al*.^19^, *	3	3	1	2	1	10
OTAS						
Mishra *et al*.^19^, *	3	1	1	2	2	9
Healey *et al*.^25^	3	2	0	1	2	8
Undre *et al*.^28^	3	2	0	1	2	8
**Incident**						
NOPE						
McCulloch *et al*.^18^	3	0	1	1	1	6
OR distraction assessment form						
Healey *et al*.^25^	2	2	0	1	1	6
Flow disruptions						
Catchpole et al.^24^	3	3	2	2	2	12
Catchpole *et al*.^20^, †	3	3	2	0	2	10
Jain *et al*.^27^	3	3	2	1	2	11
Zheng *et al*.^30^	1	1	1	1	0	4
Allers *et al*.^23^	1	1	1	1	0	4
Weigl *et al*.^29^	3	2	0	1	0	6
Interference assessment form						
Healey *et al*.^26^	2	2	1	1	1	7
**Technical**						
OCHRA						
Catchpole *et al*.^21^	3	2	0	2	1	8
Mishra *et al*.^22^	3	2	1	1	2	9
Mishra *et al*.^19^, *	3	2	0	2	1	8
OTE						
McCulloch *et al*.^18^	0	1	0	1	0	2
**Workload**						
NASA‐TLX						
Allers *et al*.^23^	0	1	0	1	0	2
Sexton *et al*.^31^	3	3	3	0	1	10
SURG‐TLX						
Weigl *et al*.^29^	3	3	0	1	1	8

* Subanalysis of observational data from McCulloch *et al*.^18^;

† subanalysis of observational data from Catchpole *et al*.^24^. NOTECHS, Oxford Non‐Technical Skills; OTAS, Observational Teamwork Assessment for Surgery; NOPE, non‐operative procedural error; OCHRA, Observational Clinical Human Reliability Assessment; OTE, Operative Technical Errors; NASA‐TLX, National Aeronautics and Space Administration – Task Load Index; SURG‐TLX, Surgery Task Load Index.

No study used the same categories of surgical flow disruption. The most frequent flow disruption categories defined across the nine studies were related to equipment (8 studies), external factors (8), communication (6), supervision/training (5), environment (5) and procedure (5) (*Tables* 2 and 3).

**Table 2:** Categories of flow disruption

Category	McCulloch *et al*.^18^	Healey *et al*.^25^	Catchpole *et al*.^24^	Catchpole *et al*.^20^, *	Jain *et al*.^27^	Zheng *et al*.^30^	Allers *et al*.^23^	Healey *et al*.^26^	Weigl *et al*.^29^	Total
Absence	X									1
Communication	X	X	X	X	X	X				6
Case‐irrelevant communication		X					X	X	X	4
Coordination	X		X	X	X					4
Supervision/training	X		X	X	X		X			5
Psychomotor error	X	X			X			X		4
Resource management	X									1
Procedural	X	X					X	X	X	5
Planning problem	X									1
Surgeon decision‐making			X		X					2
Surgeon's position change						X				1
External factors	X	X	X		X	X	X	X	X	8
External staff		X				X			X	3
Environment		X	X		X			X	X	5
Duty shift of nurses						X				1
Interference of video monitors		X						X	X	3
External resource	X								X	2
Equipment	X	X	X	X	X		X	X	X	8
Instrument changes			X		X	X				3
Robot switch					X					1
Patient factors	X		X		X					3
Safety consciousness	X									1
Vigilance/awareness	X									1

* Subanalysis of observational data from Catchpole *et al*.^24^.

**Table 3:** Explanation of flow disruption categories

Category	Explanation	Example
Absence	Team member not present	Circulating nurse out of theatre when needed
Psychomotor error	Task execution error	Sterile instrument dropped on floor
Resource management	Misjudgement of team members' ability	Surgeon leaves assistant to finish without confirming ability to do so
Procedural	Events intrinsic to the case work	Arterial clamp time not recorded
Planning problem	Known difficulty not taken into account	Difficult intubation anticipated but not prepared for consequences
Surgeon decision‐making	Technical procedural planning	Pause to determine next surgical step
External factors	Distraction from outside the operating theatre	Pager causing distraction
External staff	Disruption cause outside of surgical team	Medical student interference
Environment	Room conditions impacting flow	Incorrect room temperature
External resource problem	Organization outside the operating theatre	Essential instrument missing from standard set
Equipment	Equipment malfunction	Energy device not working
Robot switch	Robotic instrument change	Switch in controls on the robotic console
Safety consciousness	Failure to comply with safety protocols	Team member not wearing face mask
Vigilance/awareness	Failure to notice impending danger or difficulties	Failure to note significant drop in arterial pressure

Three studies included workload assessments in their methodology consisting of the National Aeronautics and Space Administration – Task Load Index (NASA‐TLX)^35^ (2) or the Surgery Task Load Index (SURG‐TLX)^36^ (1). These tools contained low to moderate evidence of validity (2–10 of 15) for the assessed outcome. Four studies also assessed technical performance using the Observational Clinical Human Reliability Assessment (OCHRA)^37^ (3) and Operative Technical Errors (OTE)^18^ (1) tools, which both have low to moderate evidence of validity (2–9 of 15).

### Team performance relative to surgical approach

Three studies^26^, ^28^, ^29^ compared team demands and/or performance in relation to a laparoscopic or open approach to abdominal procedures, reporting different results. No study compared robot‐assisted techniques with other approaches. Five studies investigated robot‐assisted techniques and found that this approach to abdominal procedures increases team demands^24^ that surgical teams were not always able to address effectively^20^, resulting in increased operating times^27^. The identification and analysis of flow disruptions can provide an evidence base for improving the efficiency and safety of robot‐assisted procedures^23^, ^31^ (*Table* S1, supporting information).

## Discussion

The primary determinants of surgical outcome are generally perceived to be the patient's condition and the performance of the individual surgeon. Once corrected for patient risk factors, surgeons' technical skills are held accountable for variation in outcome. A number of different factors are important in achieving safe and effective surgical care, including infrastructure, equipment and surgical team performance^38^. A minimally invasive surgical procedure is conducted in a sophisticated environment combining patient factors, complex equipment and a large number of individuals set to do independent and team‐based tasks.

This systematic review included 16 studies objectifying and rating surgical team performance during minimally invasive abdominal procedures. The studies were of low methodological quality, heterogeneous design, and utilized a number of different tools to objectify team performance.

In four studies, data were obtained via audiovisual recordings of the surgical environment. Despite apparent benefits of reviewing audiovisual recordings, the majority of studies collected their data through direct observation in the operating theatre. Benefits of data obtained through audiovisual recording include that data can be assessed by multiple, independent observers and incidents can be reviewed multiple times, increasing the validity and reliability of findings^39^. In addition, during direct observation the focus of the observer may decrease due to fatigue, potentially resulting in failure to record important events^40^. It is also possible that during direct observation findings are affected by the Hawthorne effect (change of behaviour in response to the awareness of being observed)^41^, ^42^. A prerequisite for accurate evaluation through review of audiovisual recordings is the quality of the recordings. Ethical considerations and potential hazards to team privacy and liability issues in case of adverse events may also be a limitation of its use^43^.

Another source of variation across the reviewed studies was the number and type of observers collecting observational data. Interobserver reliability should be quantified to guarantee the quality of observations and objectivity of rating tools used^44^.

This systematic review has demonstrated that, in the current literature, construct‐ and incident‐based team performance objectification tools are used most commonly. Construct‐based tools, including the OTAS and NOTECHS, rate a number of behavioural constructs on set Likert scales. These tools were developed for conventional approaches to surgery, providing global ratings for set constructs, and need to be validated for the identification of non‐technical skills in minimally invasive surgery. Although used by a number of studies with similar aims, studies used different categories of flow disruption, with a broad range of validity. Some variation in validity can be related to the nature of Messick's framework.

According to Reason's organizational accident model, an adverse event is preceded by a chain of individually unimportant errors and/or latent threats that in sequence lead to an adverse event or breach of patient safety^45^. Incident‐based team performance objectification methodology can provide valuable insight into these patterns and the interplay of complex minimally invasive surgical equipment.

None of the included studies was able to relate team performance to patient outcomes. This could be caused by insufficient power of individual studies. However, team performance as a determinant of morbidity and mortality is heavily biased by patient factors and technical performance. Surrogate markers for team performance could include operating time, intraoperative adverse events, and the number and duration of procedure flow disruptions. Larger, well designed studies are needed to display the true influence of minimally invasive techniques on team performance.

The major limitation of this review was the number and quality of available studies, providing insufficient data for a subgroup analysis or meta‐analysis of outcomes. The majority of studies examined a heterogeneous group of operations, providing limited validity for the identification of unique non‐technical skills related to specific procedures. Future studies should therefore analyse multiple approaches (open, laparoscopic, robot‐assisted) in relation to a single procedure, use multiple trained observers to collect data, preferably from audiovisual recordings of the surgical environment, quantify interobserver reliability, objectify team performance using incident‐based methodology with a predefined outcome set including causation and consequences of procedural flow disruptions, and analyse team performance in relation to direct (operating time, intraoperative adverse events) and indirect (patient morbidity and mortality) performance metrics. Such well designed studies are needed to gain insight into team performance demands unique to minimally invasive surgery in order to develop structured, evidence‐based training programmes that enhance patient safety and procedural flow.

## Disclosure

The authors declare no conflict of interest.

## Supporting information


**Table S1** – Characteristics of included studies
