# Supplementary material for: Systematic review of team performance in minimally invasive abdominal surgery
Source: BJS Open. 2019 Jan 30;3(3):252–9. doi: 10.1002/bjs5.50133 (PMC6551413; doi:10.1002/bjs5.50133)
Supplement: Supplementary file 1 — Table S1 – Characteristics of included studies [file BJS5-3-252-s001.docx]

**BJS5_50133**

**Systematic review of team performance in minimally invasive abdominal surgery**

**W. J. van der Vliet, S. M. Haenen, M. Solis-Velasco, C. H. C. Dejong, U. P. Neumann, A. J. Moser and R. M. van Dam**

| Table 1 – Characteristics of included studies | | | | | | |
| --- | --- | --- | --- | --- | --- | --- |
| Reference | Aim | Design | Observational methodology | Surgical procedures | Outcome measurements | Conclusion |
| Catchpole  et al. (2008)*^21^ | Investigate the relationship between teamwork, communication and technical outcome | Prospective observational,  single centre case series | Direct observation, single trained observer | 26 laparoscopic cholecystectomies & 22 carotid endarterectomies | NOTECHS, OCRA | Improved teamwork and communication may be beneficial to technical performance and patient outcomes |
| Mishra  et al. (2008)*^22^ | Investigate the relationship between non-technical performance and technical errors | Prospective observational,  single centre case series | Direct observation, single trained observer, reliability related to human factors expert | 26 laparoscopic cholecystectomies | NOTECHS, OCRA | Non-technical skills are an important component of surgical skill |
| McCulloch  et al. (2009)^18^ | Investigate the influence of non-technical skills training on teamwork, technical performance and patient outcomes | Prospective observational,  single centre, pre and post training intervention case series | Direct observation, multiple observers, including human factors expert | 58 laparoscopic cholecystectomies & 45 Carotid endarterectomies | NOTECHS, NOPE, OTE, patient outcomes | Non-technical skills training improved technical performance but did not affect patient outcomes. |
| Mishra  et al. (2009)^*19^ | Evaluation of the Oxford NOTECHS methodology in surgical teams pre- and post non-technical skills training | Prospective observational,  single centre, pre and post training intervention case series | Direct observation, multiple observers, including human factors expert, IRR quantified | 65 Laparoscopic cholecystectomies | NOTECHS, OCHRA, OTAS | The Oxford NOTECHS scale is a reliable and valid instrument for assessment of teamwork in the operating theatre |
| Healey  et al. (2008)^25^ | Evaluation of intra-operative interference by distractions and interruptions | Prospective observational,  single centre case series | Direct observation, single observer | 22 Laparoscopic cholecystectomies | OTAS, OR distraction assessment form | It is feasible to observe teamwork and to reveal the frequency and source of work interference |
| Undre  et al. (2006)^28^ | Evaluation of team performance using OTAS methodology related to procedural task completion | Prospective observational, single centre case series | Direct observation, multiple observers, including psychologist | 50 general surgery procedures, 21 laparoscopic, NOS | OTAS, procedural task checklist, patient outcomes | The OTAS methodology is a feasible tool to evaluate surgical team performance. **Surgical technique has no effect on operative time.** |
| Catchpole  et al. (2016)^24^ | Evaluate total system performance in robot assisted surgery | Prospective observational, single centre case series | Direct observation, multiple trained observers, IRR quantified | 89 robot assisted procedures (45 urologic, 30 gynaecologic, 4 cardiac) | Flow disruptions | Robot-assisted surgery increases the demands of individuals and the entire surgical team |
| Catchpole  et al. (2018) ^20^ | Sub-analysis of observational data to explore categories of flow disruptions and work demands | Prospective observational, single centre case series | Direct observation, multiple trained observers, IRR quantified | 89 robot assisted procedures (45 urologic, 30 gynaecologic, 4 cardiac) | Flow disruptions | There are a number of recurrent mismatches between work demands and the ability of humans within the **robotic surgical environment** to address them |
| Jain  et al. (2016)^27^ | Investigate the impact of flow disruptions and the relationship with resident case involvement | Prospective observational, single centre case series | Direct observation, multiple trained observers, IRR quantified | 32 robot assisted procedures (21 prostatectomies, 8 sacrocolpopexies, 3 nephrectomies) | Flow disruptions | Flow disruptions significantly increase operative duration **in robotic surgery**. Resident teaching does not increase operative duration. |
| Zheng  et al. (2008)^30^ | Identify and categorize surgical flow disruptive events | Retrospective observational, single centre case series | Postoperative audio-visual recordings review, multiple reviewers, IRR quantified | 12 laparoscopic anti-reflux procedures | Flow disruptions | Identification and categorization of flow disruptive events can increase efficiency in the operating theatre |
| Allers  et al. (2016)^23^ | Identify and categorize causes of surgical flow disruptions | Retrospective observational, single centre case series | Postoperative audio-visual recordings review, multiple reviewers, IRR quantified | 10 robot-assisted prostatectomies | Flow disruptions, NASA-TLX | Identification of surgical flow disruptions allows process modification and improvement of efficiency **in robotic surgery** |
| Cunningham et al. (2013)^17^ | Present a methodology for examining human-robot team interaction | Prospective observational, Multi-centre case series | Direct observation (2) and audio-visual recordings review (2), NOS | 4 gynaecologic robot assisted procedures | Surgical workflow, Communication | Surgical work flow analysis can be applied to study human-robot team interaction |
| Healey  et al. (2006)^26^ | Evaluation of operative interference trough interruptions and distractions | Prospective observational, single centre case series | Direct observation, multiple observers, including psychologist, IRR quantified | 50 general surgical procedures, 21 laparoscopic (cholecystectomy, fundoplication, hernia repair, appendectomy) | Interference assessment form | There is considerable distraction and interruption **due to** intrinsic and extrinsic factors during operative procedures |
| Weigl  et al. (2015)^29^ | Investigate the relationship between flow interruptions and perceived workload | Prospective observational, single centre case series | Direct observation, multiple trained observers | 56 general surgical and orthopaedic procedures (21 laparoscopic) | Interference assessment form, SURG-TLX | There is an association between number of flow interruptions and perceived workload in surgeons. **Laparoscopic surgery is associated with a higher severity of equipment related flow interruptions.** |
| Sexton  et al. (2017)^31^ | Investigate the relationship between anticipation of surgical steps and team efficiency | Retrospective observational, single centre case series | Postoperative audio-visual recordings review, multiple trained reviewers, IRR quantified | 12 robot assisted prostatectomies | Request anticipation index, NASA-TLX | Increased anticipation and team familiarity correlated with decreased requests and procedure times **in robotic surgery** |
| Guerlain  et al. (2005)^32^ | Evaluation of assessment tools for analysis of team performance, communication and situational awareness | Retrospective observational, single centre case series | Postoperative audio-visual recordings review, multiple trained reviewers | 9 Laparoscopic cholecystectomies | Laparoscopic cholecystectomy scoring tool | Assessment tools allows prospective analysis of technical judgements, team performance and communication patterns |
